# Doppler Evaluation of Fetal Cardiac Function in Gestational Diabetes Mellitus: A Scoping Review Providing Insights into Hemodynamic and Structural Alterations

**DOI:** 10.3390/jcm14165622

**Published:** 2025-08-08

**Authors:** Sophia Tsokkou, Ioannis Konstantinidis, Alkis Matsas, Evaggelia Karopoulou, Theodora Papamitsou

**Affiliations:** 1 Laboratory of Histology-Embryology, Department of Medicine, Faculty of Health Sciences, Aristotle University of Thessaloniki, 54124 Thessaloniki, Greece; thpapami@auth.gr; 2 Laboratory of Experimental Surgery and Surgical Research ‘N.S. Christeas’, Medical School, National and Kapodistrian University of Athens, 11527 Athens, Greece; amatsas@med.uoa.gr; 3 Second Department of Obstetrics and Gynecology, Aretaieion University Hospital, Medical School, National and Kapodistrian University of Athens, 76 Vasilissis Sofias Avenue, 11528 Athens, Greece; karopoulou.eva@gmail.com

**Keywords:** gestational diabetes mellitus (GDM), Doppler ultrasound, fetal cardiac function, myocardial performance index (MPI), tissue Doppler imaging (TDI), speckle-tracking echocardiography (STE), dual-gate Doppler, fetal hemodynamics, subclinical cardiac dysfunction, prenatal cardiac assessment

## Abstract

**Introduction:** Gestational diabetes mellitus (GDM) is a form of hyperglycemia that develops during pregnancy and poses risks to both the mother and fetus. In other words, it is a glucose intolerance disorder first recognized during pregnancy, specifically in the second and third trimesters, with approximately 7–14% of pregnancies worldwide being affected. **Methodology:** A systematic literature search was conducted across three major well-established databases; PubMed, Scopus, and ScienceDirect. The search was conducted with the aim of identifying the most suitable studies for the evaluation of fetal cardiac function using Doppler ultrasound techniques in pregnancies affected by GDM. **Results:** Following a comprehensive full-text assessment, 186 papers were excluded, mainly due to discrepancies in the population, unsuitable study design, publishing type, or unavailability of full-text access. Ultimately, 12 studies met all the inclusion criteria and were incorporated into the scoping review. From the studies included it was found that the conventional pulsed-wave Doppler was the most frequently used modality, assessing parameters such as the E/A ratios, myocardial performance index (MPI), and the isovolumic relaxation time (IVRT). The advanced techniques of choice included tissue Doppler imaging (TDI), speckle-tracking echocardiography (STE), dual-gate Doppler, and automated MPI. **Conclusions:** Doppler ultrasound techniques, particularly the advanced modalities like TDI and STE, provide valuable insights into fetal cardiac function in GDM pregnancies. Their integration into routine prenatal surveillance may enhance the early detection of cardiac dysfunction and inform timely clinical interventions.

## 1. Introduction

Gestational diabetes mellitus (GDM) is a form of hyperglycemia that develops during pregnancy and poses risks to both the mother and the fetus. In other words, it is a glucose intolerance disorder first recognized during pregnancy, specifically in the second and third trimesters, with approximately 7–14% of pregnancies worldwide being affected. The prevalence of GDM development is further rising along with parallel risk factors such as maternal obesity and advanced maternal age [1,2,3]. While GDM is often thought of as being a transient condition, affecting the mother only throughout the gestational period, it has now been associated with an increased risk of cardiovascular disease (CVD) later in the life of the mother. The risk extends beyond the development of type 2 diabetes to include illnesses such as coronary artery disease, myocardial infarction, stroke, and heart failure [4,5]. More specifically, a meta-analysis found that a history of GDM was associated with a 2-fold higher risk of total cardiovascular disease [6]. Another study conducted by Yu Y. et al. (2022) revealed that women with a history of GDM had a 40% increased overall CVD risk [7]. On the other hand, fetal exposure to maternal hyperglycemia leads to a cascade of metabolic and hormonal disturbances, including fetal hyperinsulinemia, oxidative stress, and altered placental signaling, which collectively contribute to cardiac remodeling and functional impairment. The fetal response to maternal hyperglycemia is determined by various factors, including the severity and timing of exposure, metabolic abnormalities, and genetic predisposition. These reactions can cause transcriptional and epigenetic alterations throughout the critical stages of heart development [8,9].

Given the nature of these cardiac alterations, Doppler-based ultrasound techniques have emerged as essential tools allowing for the non-invasive, real-time evaluation of blood flow within the fetal heart, which can reveal subtle and dynamic changes indicative of potential cardiac abnormalities [10,11,12].

### Objective

The objective of this scoping review is to systematically examine, map, and evaluate the use of Doppler-based ultrasound techniques for assessing fetal cardiac function in utero in pregnancies complicated by GDM. Specifically, this review aims to (1) identify the range of Doppler modalities and parameters applied, (2) assess their reported diagnostic value and clinical relevance, and (3) highlight methodological gaps and areas for future research. By synthesizing the current evidence, this review seeks to inform the development of evidence-based guidelines for fetal cardiac surveillance in GDM and contribute to improved perinatal cardiovascular outcomes.

## 2. Methodology

A systematic mapping of the literature on GDM-related fetal cardiac outcomes was undertaken rather than a formal meta-analysis, owing to the marked heterogeneity in study designs, diagnostic criteria, imaging modalities, and outcome measures. This study adhered to the PRISMA-ScR (Preferred Reporting Items for Systematic reviews and Meta-Analyses extension for Scoping Reviews) checklist to ensure transparent and comprehensive reporting (Table S1). Due to the nature of the study no PROSPERO registration was required for its execution.

### 2.1. Search Strategy

A systematic literature search was conducted across three major well-established databases; PubMed, Scopus, and ScienceDirect. The search was conducted with the aim of identifying the most suitable studies for the evaluation of fetal cardiac function using Doppler ultrasound techniques in pregnancies affected by GDM. We constructed a single search statement combining Medical Subject Headings (MeSH) and free-text terms with the operators AND and OR (a Boolean search strategy), including the variations highlighted in Table 1. This ensures that each concept is fully captured while minimizing irrelevant records.

### 2.2. Research Question and Eligibility Criteria

The research question was outlined more clearly using the Population, Intervention/Exposure, Comparator, Outcome (PICO) framework.

- P (Population): Fetuses of pregnant women diagnosed with gestational diabetes mellitus (GDM).
- I (Intervention/Exposure): Doppler-based fetal echocardiographic techniques.
- C (Comparator): Fetuses of pregnant women with normoglycemic pregnancies (non-diabetic controls) or within-group comparisons (well vs. poorly controlled GDM).
- O (Outcomes): Alterations in fetal cardiac structure and function, such as the myocardial performance index (MPI), E/A and E′/A′ ratios, cardiac output, ventricular wall thickness, global longitudinal strain (GLS), diastolic/systolic dysfunction indices, and predictive value for adverse perinatal outcomes.

The following eligibility criteria were created to complement the PICO framework and guide the reviewers in the screening process, ensuring that the chosen articles correctly addressed the research question.

### 2.3. Inclusion Criteria

Human studies involving pregnancies complicated by GDM;

- Use of Doppler ultrasound (spectral, tissue, or advanced techniques) for assessment of fetal cardiac function or structure;
- Original research articles, including randomized controlled trials, prospective or retrospective cohort, and case-control studies;
- Availability of full-text articles published in English.

### 2.4. Exclusion Criteria

- Studies involving only non-GDM populations;
- Animal or in vitro studies;
- Secondary literature (reviews, editorials, commentaries);
- Conference papers and abstracts;
- Articles lacking full-text access or methodological transparency.

### 2.5. Screening Process

The search process was independently performed by two reviewers as a blind process (S.T. and I.K.), coordinated at all stages of screening and data extraction to ensure methodological consistency and accuracy. Any conflicts were resolved during the second stage of the screening process by a third reviewer (A.M.).

### 2.6. Data Extraction Process

Data from the final studies included in this scoping review were extracted using structured Microsoft Excel tables. A predefined framework was employed to guide the extraction process, with the objective of capturing the essential study characteristics and outcomes. The initial author and year of publication, study design (cohort, cross-sectional, randomized controlled trial), population characteristics (including gestational age range and diagnostic criteria for GDM), type of Doppler technique used (spectral Doppler, tissue Doppler imaging, color Doppler, fetal HQ), and the key findings related to fetal cardiac structure and/or function were recorded for each study. This standardized approach ensured consistency across the dataset and facilitated the thematic synthesis and comparative analysis during the review process.

## 3. Results

The initial search identified records in PubMed (*n* = 93), Scopus (*n* = 182), and ScienceDirect (*n* = 2685), yielding a combined total of 2960 articles. After the employment of automated tools for filtering and deduplication with reference management software, 203 records were retained for screening. Five (5) duplicate entries were eliminated, and 198 distinct research papers remained for screening.

**Figure 1:**
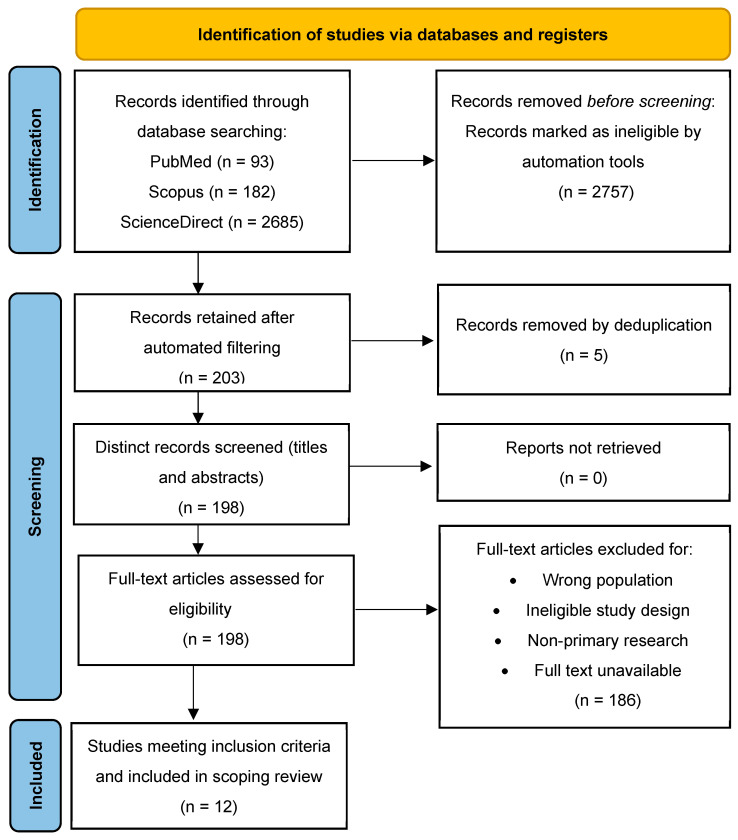
PRISMA flow diagram.

Following a comprehensive full-text assessment, 186 papers were excluded, mainly due to discrepancies in the population, unsuitable study design, publishing type, or the unavailability of full-text access. Ultimately, 12 studies met all the inclusion criteria and were incorporated into the scoping review. The PRISMA flow diagram is shown in Figure 1 and the summary table with the findings from each study is found in Table 2.

The included studies employed a range of Doppler-based ultrasound techniques for the assessment of fetal cardiac function during gestation that was complicated by the presence of GDM.

### 3.1. Conventional Pulsed-Wave Doppler

Three studies utilized pulsed-wave Doppler to evaluate the E/A ratios, the myocardial performance index (MPI), and the isovolumic relaxation time (IVRT). These parameters consistently revealed diastolic dysfunction in the fetuses of GDM mothers, with elevated MPI and reduced E/A ratios, even in the absence of structural abnormalities or poor glycemic control [13,14,15].

### 3.2. Modified and Automated MPI

Additionally, the modified MPI (Mod-MPI), which uses valve clicks for improved reproducibility, was revealed to be a sensitive predictor of adverse perinatal outcomes, with thresholds ≥ 0.52 demonstrating high sensitivity and specificity. Similarly, automated MPI (AM + MPI) provided a time-efficient, operator-independent assessment of the right ventricular function, detecting elevated MPI values in GDM fetuses despite normal umbilical artery Doppler findings [13,14,15,16].

### 3.3. Tissue Doppler Imaging (TDI)

Two studies employing tissue Doppler imaging (TDI) reported reduced E/E′ ratios, elevated LVMPI, and increased interventricular mechanical delay index (IVMDI) in GDM pregnancies, suggesting early diastolic dysfunction and mechanical dyssynchrony, with some indices (e.g., IVMDI ≥ 6.5 ms) being strongly predictive of adverse neonatal outcomes [17,18].

### 3.4. Speckle-Tracking Echocardiography (STE)

Advanced studies using speckle-tracking echocardiography (STE) demonstrated reduced atrial strain, impaired diastolic strain rates, and increased left ventricular torsion in the fetuses of diabetic mothers. These abnormalities were often present despite normal conventional Doppler findings, highlighting the superior sensitivity of STE in detecting subclinical myocardial dysfunction [19,20,21].

### 3.5. Dual-Gate Doppler

The dual-gate Doppler (DD) technique enabled the simultaneous acquisition of flow and myocardial velocity waveforms to improve temporal alignment, revealing bilateral ventricular diastolic dysfunction in the GDM fetuses and outperforming the conventional Doppler in sensitivity and reproducibility [22].

### 3.6. Aortic Isthmus Doppler

One study assessed aortic isthmus (AOI) flow using color and pulsed-wave Doppler, calculating the isthmus flow index (IFI) and the isthmus systolic index (ISI). Both indices were significantly reduced in the GDM fetuses, suggesting an early circulatory imbalance even when the peripheral Doppler indices remained normal [23].

### 3.7. Color Doppler and M-Mode

Color Doppler combined with M-mode echocardiography revealed an increased ventricular wall thickness and enhanced systolic indices (e.g., LVEF, LVFS) in the GDM-exposed fetuses. These findings support the presence of structural and functional adaptations in response to maternal hyperglycemia [24].

## 4. Discussion

### 4.1. Doppler Echocardiography—Traditional/Conventional Approach

The research conducted by Bogo et al. (2021) offers significant insights into the structural and functional cardiac modifications detected in the fetuses and neonates of mothers with gestational diabetes mellitus, despite clinically sufficient glycemic management. Doppler echocardiography, regarded as a conventional method, was employed to evaluate the myocardial thickness, myocardial performance index (MPI), and E/A ratios in the mitral and tricuspid valves during both the prenatal and postnatal periods. Hypertrophic cardiomyopathy (HPCM) was observed in 29% of the fetuses, but its incidence decreased to 6% after birth, indicating a temporary characteristic of cardiac hypertrophy. Nevertheless, functional metrics including the right and left ventricular MPI and the E/A ratios were markedly more affected during the neonatal period, suggesting that subclinical cardiac dysfunction may continue or possibly develop postnatally. The results endorse the integration of MPI and diastolic function indices as sensitive indicators in evaluating fetal heart health [13].

Similarly, an interesting study conducted by Bhorat et al. (2014) provides evidence that the fetuses of poorly managed GDM mothers demonstrate severe cardiac dysfunction, identifiable via conventional Doppler techniques. The authors demonstrated that elevated modified MPI (Mod-MPI) values and diminished mitral valve E/A ratios in the third trimester were significantly correlated with adverse perinatal outcomes, such as stillbirth, neonatal death, and NICU admission. A Mod-MPI threshold of ≥0.52 demonstrated a sensitivity of 100% and a specificity of 92% for predicting outcomes, highlighting its potential as a prognostic instrument in high-risk pregnancies. These findings underscore the diagnostic significance of functional Doppler indices in fetal surveillance and advocate for their incorporation into standard monitoring methods for pregnancies complicated by gestational diabetes mellitus [14].

In a study conducted by Pooransari et al. (2022), the embryonic cardiac function in pregnancies complicated by gestational and pregestational diabetes mellitus was examined using conventional pulsed-wave Doppler echocardiography. The study evaluated critical functional parameters, such as the left ventricular MPI, the isovolumic relaxation time (IVRT), and the E/A ratios in the mitral and tricuspid valves during the second and third trimesters. Structural measurements including interventricular septal thickness (IVST) and ventricular wall thickness (LVWT, RVWT) were assessed. The Hernandez-Andrade protocol was followed to acquire Doppler waveforms from a five-chamber view with meticulous alignment (<20° insonation angle) to capture the valve clicks for a more accurate MPI calculation. The results revealed that the diabetic group exhibited substantially elevated MPI and a prolonged IVRT, which suggests that both systolic and diastolic dysfunction were present. It is important to note that these abnormalities were observed in fetuses without myocardial hypertrophy and in mothers with excellent glycemic control, indicating that functional impairment may precede structural changes. The efficacy of standardized Doppler echocardiography as a sensitive instrument for the early detection of subclinical myocardial dysfunction in diabetic pregnancies was confirmed [15].

Considering Mod-MPI and MPI, the Mod-MPI is an adaptation of the original MPI, designed with the aim of improving the reproducibility and accuracy of fetal cardiac function assessments using Doppler ultrasound. Both are non-invasive, pulsed-wave Doppler-derived measures of global myocardial function. However, Mod-MPI utilizes the opening and closing of the mitral and aortic valves to define the isovolumetric contraction, ejection, and isovolumetric relaxation times, leading to better agreement and reduced variation compared to the original MPI [25,26,27]. The differences are summarized in Table 3.

### 4.2. Automated Right Ventricular MPI for Functional Assessment in Diabetic Pregnancies

Ding et al. (2024) examined the fetal right ventricular function during mid-to-late pregnancy utilizing automated myocardial performance index (AM+MPI) technology, contrasting the fetuses of diabetic and non-diabetic individuals. The tricuspid and pulmonary valves were recorded with synchronized inflow and outflow using color and pulsed-wave Doppler ultrasonography, respectively. The AM+MPI system minimizes operator variability by automatically identifying valve sounds to measure the isovolumic contraction time (ICT), isovolumic relaxation time (IRT), and ejection time (ET) within a normalized cardiac cycle. The right ventricular MPI was determined by dividing (ICT + IRT) by ET. The results of the study demonstrated that the MPI values of the fetuses of diabetic mothers were substantially higher than those of the controls (0.54 ± 0.05 vs. 0.48 ± 0.09, *p* < 0.05), suggesting that the general right ventricular function was impaired. It is important to note that this dysfunction was detectable in the absence of structural anomalies or aberrant umbilical artery Doppler findings. The study emphasizes the clinical utility of AM+MPI as a time-efficient, reproducible, and sensitive Doppler-based instrument for the detection of subclinical cardiac dysfunction in diabetic pregnancies [16].

### 4.3. Pulsed Tissue Doppler Imaging (TDI)

The study conducted by Hatém et al. (2008) offers a detailed evaluation of fetal diastolic function in pregnancies challenged by maternal diabetes using TDI. TDI is an advanced echocardiographic modality that enables the direct quantification of myocardial velocities throughout the cardiac cycle. The study was conducted on a total of 62 fetuses between 25 weeks gestation and term; the study compared the myocardial velocities at the mitral and tricuspid annuli among three groups: 13 fetuses of diabetic mothers with myocardial hypertrophy, 34 fetuses of diabetic mothers without myocardial hypertrophy, and 15 fetuses of mothers without diabetes. The TDI measurements were obtained from the apical four-chamber view, with sample volumes placed at the lateral mitral annulus, septal mitral annulus, and lateral tricuspid annulus. The parameters assessed included early (E′) and late (A′) diastolic myocardial velocities, systolic velocity (S′), and derived ratios such as E′/A′ and E/E′. The authors reported significantly elevated E′ and A′ velocities and reduced E/E′ ratios in the fetuses of diabetic mothers—regardless of the presence of septal hypertrophy—indicating impaired diastolic relaxation and altered ventricular compliance. The findings suggest the superior sensitivity of TDI over conventional pulsed-wave Doppler in detecting subclinical myocardial dysfunction and support its integration into fetal cardiac surveillance protocols for high-risk pregnancies such as those affected by GDM [17]. The TDI technique is used in fetal echocardiography to assess myocardial velocities and function as it provides valuable information about the heart’s systolic and diastolic performance, thus helping in the detection of potential abnormalities in fetal heart conditions. TDI is extremely useful in evaluating the myocardial motion and assessing diastolic function, which can be challenging to evaluate with conventional methods [28].

On the same note, Moghadam et al. (2024) implemented TDI to quantitatively evaluate the embryonic myocardial function and mechanical dyssynchrony in pregnancies that were complicated by overt maternal diabetes. TDI was conducted at 28 weeks gestation using an apical four-chamber view. Sample volumes were deposited at the lateral mitral annulus to derive the left ventricular myocardial performance index (LVMPI) and the early (Em) and late (Am) diastolic myocardial velocities. In addition, the temporal difference between the maximal systolic contractions of the right and left ventricles was measured to determine the interventricular mechanical delay index (IVMDI) using M-mode aligned across the interventricular septum. In comparison to the controls, the diabetic cohort exhibited substantially elevated Em, Am, LVMPI, and IVMDI values, which implies both mechanical dyssynchrony and diastolic dysfunction. It is important to note that IVMDI ≥ 6.5 ms and LVMPI ≥ 0.44 were highly predictive of adverse neonatal outcomes, with IVMDI achieving 100% specificity [18].

### 4.4. Doppler-Based Hemodynamic Assessment in GDM

Furthermore, Cai et al. (2024) contribute valuable insights into the vascular and structural cardiac adaptations in the fetuses of mothers with GDM, using conventional color Doppler ultrasound along with M-mode echocardiography. More precisely, the study assessed fetal hemodynamic parameters including the resistance index (RI), pulsatility index (PI), and systolic/diastolic ratio (S/D) in the middle cerebral artery (MCA), umbilical artery (UA), and renal artery (RA). These Doppler indices were significantly altered in the GDM group, reflecting a compensatory redistribution of fetal blood flow. In addition, M-mode echocardiography was employed for the evaluation of cardiac morphology and functionality, revealing an increased ventricular wall thickness and enhanced systolic function indices (e.g., LVEF, LVFS, RVFS) in those fetuses exposed to maternal hyperglycemia. Although the study did not utilize myocardial-specific Doppler techniques such as MPI or TDI, the broader utility of vascular Doppler surveillance in identifying subclinical cardiovascular stress in utero was emphasized. This supports the inclusion of arterial Doppler indices as complementary tools in the fetal assessment of GDM pregnancies [24]. Generally, M-mode echocardiography plays a pivotal role in fetal cardiac assessment by providing high-temporal-resolution imaging of the heart wall and valve motion, which is crucial for evaluating the fetal heart rate, rhythm, and myocardial contractility. It is especially valuable in detecting arrhythmias and assessing systolic function through measures like fractional shortening and tricuspid annular plane systolic excursion (TAPSE) [29,30].

### 4.5. Dual-Gate Doppler (DD)

The study of Hou et al. (2021) presents a comparative evaluation of fetal cardiac diastolic function in pregnancies complicated by GDM using both the conventional pulsed-wave Doppler and the emerging DD technique. The conventional Doppler assessment involved the sequential acquisition of mitral and tricuspid inflow velocities (E and A waves) and annular myocardial velocities (e′ and a′) using pulsed-wave Doppler and TDI, respectively. In contrast, the DD method enabled the simultaneous acquisition of both the flow and myocardial velocity waveforms within the same cardiac cycle by placing two independent sample volumes, one at the atrioventricular valve inflow and the other at the corresponding annulus. This approach minimized the beat-to-beat variability and improved the temporal alignment of the E/e′ ratio measurements. The study demonstrated that while both methods detected reduced E/e′ ratios in the fetuses of GDM mothers, the DD technique revealed statistically significant impairments in both the left and right ventricular diastolic function, whereas the conventional Doppler detected changes only in the right ventricle. The enhanced sensitivity and reproducibility of DD in detecting subclinical myocardial dysfunction supports its integration into fetal cardiac surveillance protocols for high-risk pregnancies [22].

### 4.6. Aortic Isthmus Doppler as an Uprising Marker of Fetal Cardiac Adaptation in GDM

Interestingly, Chen et al. (2024) employed color and pulsed-wave Doppler ultrasound to evaluate fetal aortic isthmus (AOI) hemodynamics in pregnancies complicated by GDM, offering an indirect yet physiologically meaningful assessment of fetal cardiac function. Doppler measurements included the systolic and diastolic flow velocity time integrals (S and D), the peak systolic velocity (PSV), and the systolic nadir (Ns), from which two derived indices were calculated: the isthmus flow index (IFI = (S + D)/S) and the isthmus systolic index (ISI = Ns/PSV). These indices reflect the balance of the left and right ventricular outputs and the relative vascular resistance between the cerebral and placental circulations. The study demonstrated significantly lower IFI and ISI values in the GDM group compared to the controls, suggesting early alterations in central fetal hemodynamics despite normal umbilical artery Doppler findings. Aortic isthmus Doppler can potentially be used as a sensitive adjunctive tool for detecting subclinical circulatory imbalances in GDM pregnancies, complementing the myocardial-specific Doppler techniques currently used in comprehensive fetal cardiac surveillance [23].

### 4.7. Integrated Doppler and Speckle-Tracking Assessment of Perinatal Cardiac Function in Diabetic Pregnancy

The study by Miranda et al. (2018) presented a thorough evaluation of fetal cardiac function in pregnancies complicated by maternal diabetes, employing both conventional Doppler echocardiography and a two-dimensional speckle-tracking analysis. A cohort of 129 fetuses at 30–33 weeks’ gestation was included. The researchers identified significant alterations in myocardial deformation in the diabetic group—specifically, impaired early and late diastolic strain rates in both ventricles and a reduced right ventricular global longitudinal strain (GLS)—despite the relatively preserved findings in the conventional echocardiographic parameters. These findings highlight the enhanced sensitivity of speckle-tracking in detecting subclinical functional impairment and suggest that standard Doppler indices may underestimate early cardiac dysfunction in gestational diabetes. The study supports the integration of advanced Doppler modalities, particularly speckle-tracking, into routine surveillance for high-risk pregnancies, reinforcing the objectives of this review to map and evaluate the diagnostic utility of various Doppler techniques in this context [21].

The study by Pathan et al. (2024) takes a closer look at how maternal diabetes, especially GDM, affects the developing fetal heart using cutting-edge speckle-tracking echocardiography (STE). By analyzing standard four-chamber ultrasound views, the researchers measured how the fetal left and right atria stretch and contract (strain), giving them a clearer depiction of early cardiac function. Interestingly, the fetuses exposed to maternal diabetes showed significantly lower left and right atrial strain, even though their overall heart size and function appeared normal using more traditional measures. These strain abnormalities appeared before changes in the GLS, suggesting that the atria might be more sensitive to subtle dysfunction in utero. STE reliably quantifies the intrinsic myocardial motion irrespective of the fetal position or insonation angle, making it particularly advantageous for detailed functional assessments. This study adds to the growing evidence that atrial strain could be an early marker of subclinical cardiac stress in high-risk pregnancies, and positions STE as a meaningful addition to fetal cardiac surveillance in GDM [19].

Moreover, Patey et al. (2019) conducted a prospective longitudinal study evaluating fetal and neonatal cardiac geometry and function in pregnancies affected by GDM and pre-GDM. The study employed a comprehensive echocardiographic protocol, including pulsed-wave Doppler, tissue Doppler imaging, and two-dimensional speckle-tracking echocardiography, to evaluate myocardial performance in utero and during the immediate postnatal period. Key Doppler-derived indices included the myocardial performance index (MPI′), isovolumetric contraction and relaxation times (IVCT′, IVRT′), and the E′/A′ and E/E′ ratios at the mitral, tricuspid, and septal annuli. Speckle-tracking was used to quantify the longitudinal, circumferential, and radial strain and strain rates, as well as the left ventricular torsion. Compared to controls, the fetuses and neonates of diabetic mothers exhibited altered ventricular geometry, increased wall thickness, elevated MPI′, and impaired diastolic function, with some abnormalities persisting postnatally. Notably, the LV torsion and basal radial strain rate were significantly elevated, suggesting compensatory mechanisms in response to altered loading conditions [20].

### 4.8. Strategy for Optimal Timing and Technique Selection in Fetal Cardiac Assessment of GDM Pregnancies

Based on the results of this scoping review of the available evidence, the following tiered strategy could be proposed to ensure optimal timing and technique selection for fetal cardiac assessment in GDM pregnancies, as presented in Table 4. By using pulsed-wave Doppler at the mid-trimester anatomy scan, then adding TDI and STE as the pregnancy advances, clinicians can maximize the early detection and sensitivity of subclinical cardiac alterations in GDM. Advanced techniques (dual-gate Doppler and automated MPI) should be reserved for those centers with the requisite software and expertise to further refine the risk stratification.

### 4.9. Limitations

Although this scoping review furnishes a comprehensive synthesis of Doppler-based fetal cardiac assessment in diabetic pregnancies, several caveats merit attention. First, heterogeneity in the study methodologies and in the diagnostic thresholds for gestational diabetes mellitus impeded direct comparisons across investigations. Second, most studies were observational and did not uniformly report key parameters such as maternal glycemic control or postnatal cardiac outcomes. Third, our restriction to English language publications and a limited set of databases may have led to the omission of relevant research. Finally, by design, this scoping review did not undertake a formal critical appraisal of study quality. Nevertheless, despite these limitations, the findings illuminate the emerging trends and identify critical gaps, thereby guiding future research efforts.

### 4.10. Future Directions

Future research should aim to prospectively validate a structured, tiered surveillance protocol for fetal cardiac assessment in GDM pregnancies. Ideally, this protocol begins with the conventional pulsed-wave Doppler at the mid-trimester anatomy scan (24–28 weeks) to quantify the E/A ratios, isovolumic contraction and relaxation times, and global MPI, all of which have been shown to unmask early diastolic dysfunction. In the early third trimester (28–32 weeks), tissue Doppler imaging should be added to measure the annular E′/A′ and E/E′ ratios and the interventricular mechanical delay, thereby detecting subclinical diastolic impairments that may not be apparent using the conventional Doppler alone. Finally, speckle-tracking echocardiography performed at 30–34 weeks can quantify the atrial and ventricular strain and diastolic strain rates, offering the greatest sensitivity for detecting subtle systolic and diastolic abnormalities. Where available, dual-gate Doppler to enable simultaneous flow and tissue velocity measurements, and automated MPI algorithms should be integrated to further reduce operator dependency and improve the temporal alignment of key indices.

In parallel, future studies should establish gestational age- and GDM-specific normative percentiles for pulsed-wave, tissue Doppler, speckle-tracking, and automated MPI parameters. Complementary research should evaluate the use of mid- to late-gestation umbilical cord NT-proBNP levels as a biochemical trigger for targeted fetal echocardiographic screening, refining risk stratification in poorly controlled GDM. Longitudinal cohorts that correlate the prenatal Doppler and strain indices with postnatal cardiac structure, function, and early childhood cardiovascular risk are also essential. Finally, cost-effectiveness analyses comparing standard obstetric care to this multimodal surveillance approach will guide implementation and inform future guideline recommendations, with the ultimate goal of improving cardiovascular outcomes in the offspring of women with GDM.

## 5. Conclusions

Doppler-based ultrasound techniques, including pulsed-wave Doppler, tissue Doppler imaging, speckle-tracking echocardiography, dual-gate Doppler, and automated myocardial performance index, reliably detect subclinical structural and functional cardiac alterations in fetuses exposed to gestational diabetes mellitus. These modalities reveal diastolic dysfunction, myocardial performance impairments, and early hemodynamic imbalances even in pregnancies with optimal glycemic control, underscoring their value for enhanced fetal surveillance. Implementing a tiered assessment strategy that progresses from the conventional Doppler at mid-trimester to advanced deformation and automated methods in the third trimester may improve risk stratification and guide timely interventions. The prospective validation of standardized protocols, establishment of GDM-specific normative ranges, and longitudinal correlations with postnatal cardiovascular outcomes are essential next steps to translate these findings into clinical practice.

## Figures and Tables

**Table 1:** Search strategy and terms used in PubMed, Scopus, and ScienceDirect.

Search Strategy and Terms Used in PubMed, Scopus, and ScienceDirect
((“fetal” [All Fields] OR “fetally” [All Fields] OR “fetals” [All Fields] OR “fetus” [MeSH Terms] OR “fetus” [All Fields] OR “fetal” [All Fields] OR “fetal” [All Fields]) AND (“doppler” [All Fields] OR “doppler s” [All Fields] OR “dopplers” [All Fields]) AND (“diabetes, gestational” [MeSH Terms] OR (“diabetes” [All Fields] AND “gestational” [All Fields]) OR “gestational diabetes” [All Fields] OR (“gestational” [All Fields] AND “diabetes” [All Fields] AND “mellitus” [All Fields]) OR “gestational diabetes mellitus” [All Fields]) AND (“fetal heart” [All Fields] OR “fetal heart” [MeSH Terms] OR (“fetal” [All Fields] AND “heart” [All Fields]) OR “fetal heart” [All Fields])) AND (case reports [Filter] OR clinical study [Filter] OR clinical trial [Filter] OR clinical trial phase i [Filter] OR clinical trial phase ii [Filter] OR clinical trial phase iii [Filter] OR comparative study [Filter] OR controlled clinical trial [Filter] OR multicenter study [Filter] OR observational study [Filter] OR randomized controlled trial [Filter])

**Table 2:** Summary of Doppler-based studies evaluating fetal cardiac structure and function in diabetic pregnancies.

Authors	Year	Study Design	Population	GDM Diagnostic Criteria	Gestational Age (GA)	Doppler Technique	Parameters Measured	Key Findings
Bogo et al.	2021	Prospective descriptive observational study	48 fetuses and newborns of insulin-treated GDM mothers; singleton pregnancies; no malformations	ADA: FPG ≥ 92 mg/dL, 1 h >180 mg/dL, 2 h ≥ 153 mg/dL	Fetal exams: 22–37 weeks Neonatal: first 2 months	Doppler and M-mode (Philips EnVisor C, S4 probe, 2–4.2 MHz)	Myocardial thickness, LV and RV myocardial performance index (MPI), Shortening fraction, mitral and tricuspid valve E/A ratios	Hypertrophic cardiomyopathy (HPCM): 29% fetal vs. 6% postnatal (*p* = 0.006), RVMPI altered: 12.5% vs. 54.2% (*p* < 0.001), LVMPI altered: 27.1% vs. 60.4% (*p* = 0.001), Mitral E/A altered: 6.3% vs. 50% (*p* < 0.001), Tricuspid E/A altered: 0% vs. 27.1% (*p* < 0.001)
Bhorat et al.	2014	Prospective cross-sectional study	29 women with poorly controlled GDM on insulin (3rd trimester), matched with 29 normal pregnancies (controls); singleton, no anomalies	Not explicitly stated; all were insulin-requiring with poor glycemic control	Assessment: mean GA = 35 weeks Delivery: approximately 38.4 weeks in GDM group	Doppler echocardiography using Mod-MPI and E/A ratios via GE Voluson E8 or Siemens Antares systems	Modified myocardial performance index (Mod-MPI), E/A ratio (mitral valve), ICT, IRT, ET, placental resistance markers (UA RI, MCA RI, DV PI)	Mod-MPI significantly increased in GDM fetuses: 0.59 vs. 0.38, *p* < 0.0001 E/A ratio lower: 0.65 vs. 0.76, Adverse outcomes in 17/29 GDM fetuses: NICU admission, tachypnea, acidosis, cardiomyopathy, stillbirths, Mod-MPI ≥ 0.52: Sensitivity 100%, specificity 92% for predicting adverse outcomes, no adverse outcomes in control group, Doppler cardiac data not used in management; CTG and UA Doppler alone missed critical compromise
Pooransari et al.	2022	Observational case-control study	100 singleton pregnancies: 50 diabetic (40 GDM, 10 overt DM) and 50 controls; 22 with poor glycemic control	GDM diagnosed using FBS ≥ 92 mg/dL or OGTT ≥ 153 mg/dL	2nd–3rd trimester (18–40 weeks); median: 22 weeks	Fetal echocardiography with pulsed Doppler and structural imaging on GE Vivid E8	Left MPI, isovolumic relaxation time (IVRT), E/A ratios (mitral, tricuspid), LVWT, RVWT, IVST	Higher left MPI in diabetics: 0.53 ± 0.15 vs. 0.43 ± 0.09 (*p* < 0.05), IVRT > 41 ms more frequent in diabetics, LVWT, RVWT, and IVST significantly greater in diabetic group (*p* < 0.05), structural abnormalities (e.g., IVST > 4 mm, LVWT > 4.9 mm) occurred only in diabetic group, MPI elevated even in diabetics with good glycemic control, MPI and RVWT were strong predictors of adverse fetal outcomes using decision tree models
Ding et al.	2024	Prospective observational study	102 pregnant women (85 healthy, 17 with diabetes); singleton, no anomalies	Not explicitly stated; clinically diagnosed DM	20–38 weeks (mid-to-late pregnancy)	Automated myocardial performance index (AM+MPI) using Samsung WS80A ultrasound (RV inflow/outflow Doppler)	Right ventricular MPI, isovolumic contraction time (ICT), isovolumic relaxation time (IRT), ejection time (ET)	RV MPI significantly higher in diabetic fetuses: 0.54 ± 0.05 vs. 0.48 ± 0.09 (*p* < 0.05), IRT positively correlated with gestational age (IRT = 29.78 + 0.49 × GA; *p* < 0.05), no significant variability in MPI, ET, or ICT with GA, IRT showed linear increase with GA, AM+MPI provides a stable, efficient, and reproducible method for assessing fetal RV function, findings support its potential for early detection of functional abnormalities in diabetic pregnancies
Hatém et al.	2008	Cross-sectional observational study	62 fetuses total: 47 from diabetic mothers (13 with, 34 without myocardial hypertrophy), 15 controls from non-diabetic mothers	ADA, WHO, and Brazilian Diabetes Society guidelines used; details not fully specified	25 weeks to term (mean 32 weeks)	Tissue Doppler imaging (Siemens Aspen, Philips HP Sonos 5500, GE Vivid III) across mitral, septal, and tricuspid annuli	E′, A′, and S′ velocities at mural/anterior mitral annulus and tricuspid annulus, E/A and E/E′ ratios, conventional mitral/tricuspid inflow velocities	E′ and A′ velocities significantly higher in diabetic fetuses vs. controls (*p* < 0.05), lower E/E′ ratios in diabetic fetuses at both valves despite normal inflow E/A ratios, diastolic dysfunction evident independently of myocardial hypertrophy, differences in velocity patterns based on annular location (septal < mural < lateral),tissue Doppler revealed abnormalities missed by conventional inflow Doppler, findings suggest TDI detects early diastolic dysfunction—even before structural hypertrophy develops
Moghadam et al.	2024	Case-control study	26 women with overt diabetes (pre-gestational) vs. 26 healthy controls; singleton pregnancies	Overt DM diagnosed pre-pregnancy or early gestation	Fetal exams at 18–22 weeks and 28 weeks; neonatal assessment at 1 week postnatal	Tissue Doppler echocardiography (Philips Affiniti 50 with C6-2 MHz and S8-3 MHz probes)	Em, Am, Em/Am, LVMPI, IVMDI; dyssynchrony; neonatal outcomes (birthweight, Apgar, hospitalization, echo)	Significantly higher Em (*p* = 0.007), Am (*p* < 0.001), LVMPI (*p* = 0.003), IVMDI (*p* = 0.026) in diabetic group, no difference in Em/Am ratio (*p* = 0.264), dyssynchrony present in 30.8% of diabetic fetuses vs. 0% in controls (*p* = 0.004), adverse neonatal outcomes more frequent in diabetic group (46.2% vs. 7.7%; RR: 8.8, *p* = 0.009), only LVMPI (*p* = 0.034) and IVMDI (*p* < 0.001) were predictive of neonatal complications, IVMDI ≥ 6.5 ms: 67% sensitivity, 100% specificity (RR: 24.5), LVMPI ≥ 0.44: 75% sensitivity, 79% specificity (RR: 11), dyssynchrony and worse outcomes only observed in diabetic subgroup with poor glycemic control
Cai et al.	2024	Observational case-control study	110 third-trimester women (55 GDM, 55 controls); singleton pregnancies, no chromosomal abnormalities or malformations	FBG ≥ 5.6–5.8 mmol/L or OGTT thresholds exceeded: 1 h ≥ 10.3, 2 h ≥ 8.6, 3 h ≥ 6.7 mmol/L (Chinese clinical thresholds)	Third trimester; ~36.8 weeks average	Color Doppler ultrasound using Hitachi HI VISION Avius and GE Voluson 730ProV (3–5 MHz probe)	RI, PI, S/D of MCA, UA, and RA, cardiac parameters: MVA, TVA, AVA, PVA, LVDd, LVDs, RVDd, RVDs, LVWT, RVWT, LVEF, LVFS, RVFS, maternal serum Cys C and Hcy levels, neonatal outcomes: Apgar, NICU admission, macrosomia, hypoxia-related conditions	MCA RI, PI, S/D decreased; UA and RA RI, PI, S/D increased in GDM fetuses (all *p* < 0.05), cardiac structures (MVA, TVA, AVA, PVA) and LV/RV wall thicknesses significantly larger in GDM group, higher LVEF, LVFS, RVFS in GDM group, Cys C: 1.35 mg/L vs. 0.69 mg/L; Hcy: 19.88 vs. 10.17 μmol/L, adverse pregnancy outcomes (APO) rate: 25.45% in GDM vs. 10.91% in controls, ROC: AUCs for MCA S/D = 0.875, UA RI = 0.863, RA S/D = 0.850; Cys C = 0.753, logistic regression confirmed significant associations between these markers and APO risk (*p* < 0.05)
Hou et al.	2021	Prospective cross-sectional study	111 singleton pregnancies (56 GDM, 55 controls); 24–30 weeks gestation; matched GA; excluded fetal/maternal conditions	IADPSG criteria: OGTT ≥ 5.1 (0 h), ≥10.0 (1 h), ≥8.5 mmol/L (2 h); per Chinese GDM guidelines	24–30 weeks GA	Dual-gate Doppler (DD) + conventional pulsed Doppler and tissue Doppler imaging (TDI) (ALOKA F75 system)	E/A ratio, e′/a′ ratio, E/e′ ratio (mitral and tricuspid), Peak E, A, e′, a′ velocities using both methods	E/e′ ratios significantly lower in GDM group for both LV and RV using DD method (*p* = 0.036 for LV, *p* = 0.01 for RV), RV E/e′ also significantly reduced using conventional Doppler (*p* = 0.001), RV showed greater sensitivity to dysfunction than LV, tricuspid e′ velocity increased in GDM group (*p* = 0.037), suggesting adaptation to reduced compliance, no significant differences in E/A or e′/a′ ratios between groups, well vs. poorly controlled GDM: no significant differences in DD measures suggesting that even well-controlled hyperglycemia affects fetal diastolic function, DD method shown to be feasible, reproducible, and more sensitive than conventional Doppler
Chen et al.	2024	Cross-sectional observational study	94 singleton pregnancies: 47 with GDM, 47 healthy controls (matched for GA and age)	OGTT (24–28 weeks): FPG ≥ 5.1 mmol/L, 1 h ≥ 10.0 mmol/L, 2 h ≥ 8.5 mmol/L	Mean GA: 32.8 weeks	Color Doppler of aortic isthmus (GE Voluson E8/S10, 1–5 MHz probe)	Systolic and diastolic flow velocity time integrals (S, D), peak systolic velocity (PSV), systolic nadir (Ns), IFI = (S+D)/S, ISI = Ns/PSV	D, Ns, IFI, and ISI were significantly lower in GDM group (e.g., D: 2.39 cm vs. 2.76 cm, *p* = 0.015; IFI: 1.24 vs. 1.27, *p* = 0.042), no significant differences in umbilical artery Doppler (PSV, EDV, PI, S/D), IFI and ISI declined with gestational age in GDM fetuses, especially ISI showing a linear downward trend, suggests earlier hemodynamic changes in aortic arch circulation than in umbilical artery, combining IFI and ISI may improve prenatal assessment of fetal circulatory impairment in GDM
Miranda et al.	2017	Cross-sectional observational study	129 fetuses: 76 exposed to maternal diabetes (69 GDM, 7 pregestational); 53 controls with normal fetal hearts	International criteria: FPG or 75g OGTT between 24–28 weeks (details not fully specified)	30–33 weeks	Conventional echocardiography + 2D speckle-tracking echocardiography (Vivid E95 system, GE Healthcare)	IVS thickness, cardiac output (CO), shortening fraction (SF), MAPSE/TAPSE, mitral/tricuspid E/A ratio, modified MPI, LV/RV global longitudinal strain (GLS), early and late diastolic strain rates (SRe, SRa), segmental and global deformation rates	IVS significantly thicker in maternal diabetes (MD) group (*p* < 0.001), lower LV cardiac output in MD fetuses (*p* < 0.05), no significant differences in conventional diastolic/systolic function markers, lower SRe and SRa in both ventricles, indicating diastolic dysfunction (*p* < 0.05), RV GLS significantly lower in MD fetuses: −13.67% vs. −15.52% (*p* < 0.05), diastolic dysfunction occurred independently of septal hypertrophy, speckle-tracking detected subclinical biventricular dysfunction missed by conventional methods, maternal age independently predicted worse GLS in MD group, non-medicated GDM subgroup had more impaired function than insulin/metformin-treated group
Pathan et al.	2024	Prospective observational study	151 singleton pregnancies: 104 with maternal diabetes (GDM, T1DM, T2DM), 47 healthy controls; matched for maternal age and GA	ADIPS criteria: OGTT with FPG ≥ 5.1 mmol/L, 1 h ≥ 10.0 mmol/L, 2 h ≥ 8.5 mmol/L (Australia)	Mean approximately 28.6 weeks	Speckle-tracking echocardiography (GE Voluson, RAB6-RS probe; TomTec Image Arena software)	Left atrial strain (LAS), right atrial strain (RAS), left atrial area (LAA), right atrial area (RAA), septal thickness (IVS), LV GLS and RV free wall strain (RV FWS)	LAS significantly reduced in DM group: 28.8 ± 8.8% vs. 33.3 ± 10.4% (*p* = 0.007); DM independently predicted reduced LAS after multivariable adjustment, RAS also reduced: 27.7 ± 10.4% vs. 31.8 ± 10.3% (*p* = 0.02); RV FWS and fetal weight were main determinants, IVS significantly thicker in DM group (*p* = 0.001), no significant difference in LV GLS or birthweight. Suggests subclinical atrial dysfunction occurs in diabetic fetuses even when conventional markers appear normal
Patey et al.	2019	Prospective longitudinal study	75 term pregnancies (54 normal; 21 diabetic: 14 GDM on metformin, 7 PGDM on insulin); singleton, no anomalies	UK NICE guidelines for diabetes in pregnancy; all had appropriate glycemic control	Term (around 38–40 weeks)	B-mode, M-mode, PW Doppler, tissue Doppler imaging (TDI), and speckle-tracking imaging (STI) using GE Vivid E9 (fetal and neonatal exams)	Cardiac geometry: chamber dimensions, sphericity index, wall thickness, myocardial performance indices (LV-MPI’, RV-MPI’), diastolic function: E′/A′, E/E′ ratios, IVRT’, systolic function: CO, S′ velocities, IVCT’, LV torsion, myocardial deformation: longitudinal, circumferential, radial strain and strain rate, left ventricular twist/torsion across fetal and neonatal stages	Diabetic fetuses showed increased RV sphericity index, thicker IVS and ventricular walls, reduced LV dimensions, and lower LV CO, elevated myocardial strain (e.g., basal circumferential/radial) in fetuses, decreased longitudinal strain rates postnatally, postnatal persistence of altered cardiac geometry and dysfunction, especially LV torsion, IVRT′, MPI′ and E′/A′ ratios, tricuspid regurgitation more common in diabetic neonates (48% vs. 6%), fetal/neonatal changes likely reflect adaptive response to fetal hypoxemia, metabolic derangement and altered loading conditions at delivery

**Table 3:** Myocardial performance index (MPI) versus modified myocardial performance index (Mod-MPI).

Feature	Myocardial Performance Index (MPI)	Modified Myocardial Performance Index (Mod-MPI)
Definition	A Doppler-derived index assessing global cardiac function via systolic and diastolic time intervals	A refined version of MPI using valve clicks for more precise time interval measurement
Formula	(ICT + IRT)/ET	Same formula: (ICT + IRT)/ET
Landmark Identification	Based on waveform morphology (e.g., mitral inflow and aortic outflow)	Based on valve opening and closing clicks (mitral and aortic valves)
Reproducibility	Moderate; operator-dependent	Higher reproducibility due to clearer anatomical landmarks
Clinical Use	Used in both adult and fetal cardiology	Primarily used in fetal echocardiography
Sensitivity to Dysfunction	Good for detecting global dysfunction	More sensitive to subtle or early-stage fetal cardiac dysfunction
Preferred In	General cardiac assessments	High-risk pregnancies (e.g., GDM, IUGR) where standardization and precision are critical

**Table 4:** Strategy for optimal timing and technique selection for fetal cardiac assessment in GDM pregnancies.

Gestational Weeks	Diagnostic Tool	Assessment	Rationale—Reference
Mid-trimester screening (24–28 weeks’ gestation)	Conventional pulsed-wave Doppler	Measure E/A ratios, isovolumic contraction time (ICT), isovolumic relaxation time (IRT) and myocardial performance index (MPI).	Bhorat et al., 2014 [14] and Pooransari et al., 2022 [15] demonstrated that diastolic dysfunction often appears by 24–28 weeks, even before septal hypertrophy is evident.
Early third-trimester follow-up (28–32 weeks’ gestation)	Tissue Doppler imaging	Assess E′/A′ and E/E′ ratios and interventricular mechanical delay index (IVMDI).	Hatém et al., 2008 [17] and Moghadam et al., 2024 [18] showed that TDI at this stage reliably unearths early diastolic impairment and dyssynchrony.
Advanced deformation analysis (30–34 weeks’ gestation)	Speckle-tracking echocardiography (STE)	Quantify global longitudinal strain (GLS), atrial strain, and diastolic strain rates.	Miranda et al., 2018 [21] and Pathan et al., 2024 [19] found that STE is most sensitive for detecting subclinical systolic and diastolic dysfunction when applied in the early third trimester.
Supplementary modalities (any time after 20 weeks where available)	Dual-gate Doppler for simultaneous E/e′ ratio measurement to minimize beat-to-beat variability		
	Automated MPI for operator-independent, efficient assessment of right ventricular function from mid-trimester onward.		

## Supplementary Materials

The following supporting information can be downloaded at: https://www.mdpi.com/article/10.3390/jcm14165622/s1, Table S1: PRISMA-ScR Table. Ref. [31] is cited in Supplementary Materials.
